# Honey Botanical Origin Authentication Using HS-SPME-GC-MS Volatile Profiling and Advanced Machine Learning Models (Random Forest, XGBoost, and Neural Network)

**DOI:** 10.3390/foods15020389

**Published:** 2026-01-21

**Authors:** Amir Pourmoradian, Mohsen Barzegar, Ángel A. Carbonell-Barrachina, Luis Noguera-Artiaga

**Affiliations:** 1 Department of Food Science and Technology, Faculty of Agriculture, Tarbiat Modares University, Tehran P.O. Box 14115-336, Iran; amirppoormoradi2020@gmail.com; 2 Grupo de Investigación “Calidad y Seguridad Alimentaria”, Instituto de Investigación e Innovación Agroalimentaria y Agroambiental (CIAGRO-UMH), Universidad Miguel Hernández de Elche, Carretera de Beniel, km 3.2, 03312 Alicante, Spain; angel.carbonell@umh.es (Á.A.C.-B.); lnoguera@umh.es (L.N.-A.)

**Keywords:** honey authentication, chromatography, volatile compounds, GC—MS, botanical origin, chemometrics, neural network

## Abstract

This study develops a comprehensive workflow integrating Headspace Solid-Phase Microextraction Gas Chromatography–Mass Spectrometry (HS-SPME-GC-MS) with advanced supervised machine learning to authenticate the botanical origin of honeys from five distinct floral sources—coriander, orange blossom, astragalus, rosemary, and chehelgiah. While HS-SPME-GC-MS combined with traditional chemometrics (e.g., PCA, LDA, OPLS-DA) is well-established for honey discrimination, the application and direct comparison of Random Forest (RF), eXtreme Gradient Boosting (XGBoost), and Neural Network (NN) models represent a significant advancement in multiclass prediction accuracy and model robustness. A total of 57 honey samples were analyzed to generate detailed volatile organic compound (VOC) profiles. Key chemotaxonomic markers were identified: anethole in coriander and chehelgiah, thymoquinone in astragalus, p-menth-8-en-1-ol in orange blossom, and dill ester (3,6-dimethyl-2,3,3a,4,5,7a-hexahydrobenzofuran) in rosemary. Principal component analysis (PCA) revealed clear separation across botanical classes (PC1: 49.8%; PC2: 22.6%). Three classification models—RF, XGBoost, and NN—were trained on standardized, stratified data. The NN model achieved the highest accuracy (90.32%), followed by XGBoost (86.69%) and RF (83.47%), with superior per-class F1-scores and near-perfect specificity (>0.95). Confusion matrices confirmed minimal misclassification, particularly in the NN model. This work establishes HS-SPME-GC-MS coupled with deep learning as a rapid, sensitive, and reliable tool for multiclass honey botanical authentication, offering strong potential for real-time quality control, fraud detection, and premium market certification.

## 1. Introduction

Honey is a naturally derived product that has played a significant role in human nutrition since antiquity, primarily due to its nutritional and medicinal properties [1]. Although primarily composed of sugars, honey contains a diverse range of bioactive components, including enzymes, amino acids, organic acids, carotenoids, vitamins, minerals, and aromatic compounds. Its chemical profile and sensory attributes—color, aroma, and flavor—are predominantly influenced by botanical and geographical origin, while secondary factors such as packaging and storage conditions further modulate its quality [2].

In 2023, the global honey market reached approximately USD 9.3 billion, driven by rising consumer preference for natural, minimally processed sweeteners. Honey is widely recognized as a functional food due to its antioxidant, antimicrobial, and anti-inflammatory activities, contributing to cardiovascular support, immune enhancement, and accelerated wound healing [3].

Authenticity verification is essential because monofloral honey from specific botanical and geographic origins typically commands a higher market value. Authenticity involves both (i) ensuring purity by avoiding adulteration with sugars or lower-quality honey and (ii) verifying that labeling accurately represents the product’s origin. Traditional authentication approaches—melissopalynology, sensory evaluation, and physicochemical tests—are often labor-intensive, require expert knowledge, and exhibit limitations, particularly when pollen content fails to reflect floral origin. To address these issues, modern analytical tools, including spectroscopic methods (IR, Raman, and NMR), isotopic analyses, biosensors, and chromatographic techniques, are increasingly used, often in combination with chemometric methods [4].

Chromatographic techniques, especially liquid chromatography (LC) and gas chromatography (GC), provide chemical fingerprints that facilitate the identification of biomarkers such as phenolic acids, flavonoids, alkaloids, and certain aromatic aldehydes and furans [5].

GC-MS is particularly advantageous for VOC analysis due to its sensitivity, robustness, and reproducibility [6]. HS-SPME is the preferred extraction method because it enables solvent-free, minimally invasive pre-concentration of volatiles without thermal degradation [7].

Previous studies have demonstrated the usefulness of chemometrics for honey authentication. Corvucci et al. (2015) found strong agreement between PCA-based classification and traditional melissopalynology [8]. Karabagias et al. (2020) achieved >89% classification accuracy for monofloral honeys using only nine volatile compounds [9]. Tian et al. (2024) successfully applied LC-QTOF-MS with chemometrics for non-targeted discrimination of multiple honey origins [5]. These results highlight a rapid, straightforward, and robust analytical strategy for honey chemical fingerprinting, facilitating marker identification and the development of advanced predictive models for botanical origin authentication.

While the combination of HS-SPME-GC-MS with traditional chemometric methods (such as PCA, LDA, and OPLS-DA) is well-established and widely applied for the discrimination of honey botanical origins, the integration of advanced supervised machine learning algorithms—particularly the direct comparison of ensemble methods (RF, XGBoost) and NNs—for improved multiclass classification accuracy has received limited attention. The present work addresses this by developing and benchmarking three machine learning models on volatile profiles from five botanically diverse honeys (including less commonly studied sources such as chehelgiah and astragalus)

## 2. Materials and Methods

### 2.1. Honey Sample Preparation

Nineteen honey samples from five botanical origins—coriander (n = 4), orange blossom (n = 4), chehelgiah (n = 4), astragalus (n = 4), and rosemary (n = 3)—were obtained from farms in Spain and Iran (Supplementary Materials), yielding a total of 57 samples (three technical/instrumental replicates per batch). Two grams of each honey sample were dissolved in 8 mL of deionized water and vortexed until a clear solution formed. Samples were stored in sealed containers at room temperature before analysis.

### 2.2. HS-SPME Extraction

Volatile compound extraction followed the method of Quintanilla-López et al. (2022) [4] with modifications. Ten milliliters of each honey solution was transferred to 20 mL SPME vials, leaving a 10 mL headspace. A 1 cm length Divinylbenzene/Carboxen/Polydimethylsiloxane (DVB/CAR/PDMS) fiber (50/30 µm) was used for adsorption. Samples were exposed to the fiber for 30 min at 60 °C with agitation (500 rpm). Extraction was performed using a Shimadzu AOC-6000 Plus autosampler (Shimadzu Corporation, Kyoto, Japan) for precise temperature and agitation control.

### 2.3. GC-MS Analysis

GC analysis was conducted using an SLB-5 MS capillary column (30 m × 0.25 mm × 0.25 µm; Teknokroma, Barcelona, Spain). The oven program was 50 °C (initial), 3 °C/min to 130 °C, then 8 °C/min to 205 °C, followed by 4 °C/min to 250 °C. Helium (99.9999%) was used as the carrier gas at 1 mL/min. Injection was performed in splitless mode at 250 °C. A Shimadzu TQ8040 NX mass spectrometer (Shimadzu Corporation, Kyoto, Japan) operated at temperatures of 230 °C for the ion source and 150 °C for the quadrupole, with 70 eV electron ionization. The scanning range was *m*/*z* 50–500 at 2 scans/s, with a solvent delay of 5.5 min. Volatile compounds were identified based on (i) calculating retention indices using a C8–C24 alkane series (Sigma-Aldrich, Steinheim, Germany); (ii) comparison with retention times of analytical standards; (iii) spectral matching with reference libraries [6].

### 2.4. Chemometric Analysis

The chemometric analyses were conducted on a data matrix of dimensions 57 × 40, where rows represent individual samples (including technical replicates) and columns correspond to the relative peak areas (%) of the 40 identified VOCs (see Table 1 and Table 2). These features were derived from integrated peak areas normalized to total ion current, rather than raw chromatographic signals, to emphasize origin-specific markers and minimize instrumental artifacts. PCA was employed to visualize clustering among botanical origins. For classification, RF, XGBoost, and NN models were implemented in Python 3.7 [10].

Data preprocessing included mean imputation for missing values, z-score standardization, and a stratified 70/30 train–test split performed at the independent batch level (n = 19 batches) to avoid data leakage. Missing values (~5–10% of entries) occurred due to compounds below LOD, unreliable integration at trace levels, or biological absence in specific botanical classes (see Table 2). These were imputed using column-wise mean substitution, a standard conservative approach in chemometrics for low-missingness datasets that preserves data structure without bias. Sensitivity tests verified negligible impact on PCA clustering or model performance. All technical replicates (three per batch) from the same independent honey batch were kept together in either the training or test set.

These classification models were utilized to differentiate among five distinct types of botanical honey.

RF (RandomForestClassifier): n_estimators = 100, criterion = ‘gini’) and random_state = 42.

XGBoost (XGBClassifier): use_label_encoder = False, eval_metric = ‘mlogloss’, random_state = 42. No hyperparameter tuning was performed beyond defaults.

NN (MLPClassifier): One hidden layer (100 neurons), max_iter = 500, random_state = 42. Default activation and solver settings were retained.

Performance metrics included accuracy, precision, recall, F1-score, sensitivity, and specificity [11].

## 3. Results

### 3.1. VOC Fingerprinting

HS-SPME-GC-MS identified 40 VOCs across samples, belonging to hydrocarbons, alcohols, aldehydes, ketones, acids, esters, heterocycles, terpenes/terpenoids, amines, nitriles, and phenolic compounds. Table 1 and Table 2 summarize the compound identities and distributions.

Anethole, a phenylpropanoid derivative with a characteristic anise-like aroma, was the dominant marker in coriander honey. This compound is biosynthetically derived from the shikimate pathway in Coriandrum sativum. Its presence confirms monofloral origin and aligns with previous findings [12]. The presence of anethole at such elevated levels not only validates botanical authenticity but also contributes to the honey’s potential antioxidant and antimicrobial properties.

Thymoquinone emerged as the major VOC reflecting the known phytochemistry of astragalus spp. [13]. This compound, known for its anti-inflammatory, antioxidant, and anticancer activities, is likely translocated from the plant’s root or aerial parts via nectar.

p-menth-8-en-1-ol was the hallmark compound of orange blossom honey, reflecting the rich monoterpenoid metabolism of Citrus sinensis and Citrus aurantium blossoms. This compound contributes to the fresh, citrusy, and slightly minty aroma typical of orange blossom honey. Its high concentration confirms the authenticity of the floral source and distinguishes this honey from others lacking citrus-derived volatiles [14]. The presence of this stereoisomer further supports the specificity of nectar transfer, as it is rarely found in significant amounts in non-citrus honeys.

The bicyclic furan derivative dill ester dominated the VOCs of rosemary honey. While not commonly reported as a primary marker, this compound is likely a degradation product or derivative of verbenone or camphor—key constituents of Rosmarinus officinalis. Its prevalence suggests active biochemical transformation during honey ripening or storage. This unique furanoid structure contributes to the warm, herbaceous, and slightly camphoraceous aroma of rosemary honey and serves as a novel candidate marker for authentication, particularly when combined with linalool and 1,8-cineole in multivariate models [15].

### 3.2. Chemometric Analysis

**Figure 1:**
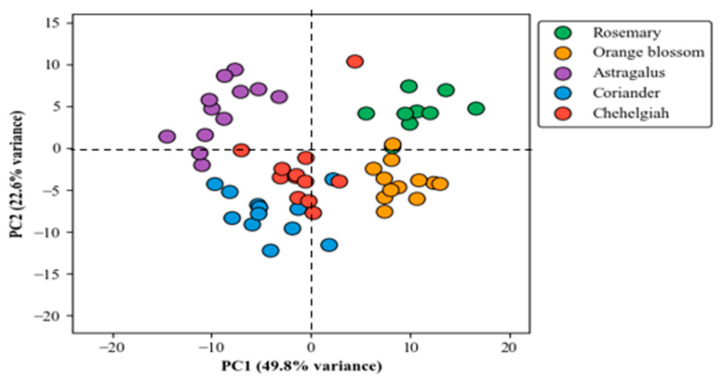
PCA plot showing clustering of different botanical honeys.

**Figure 2:**
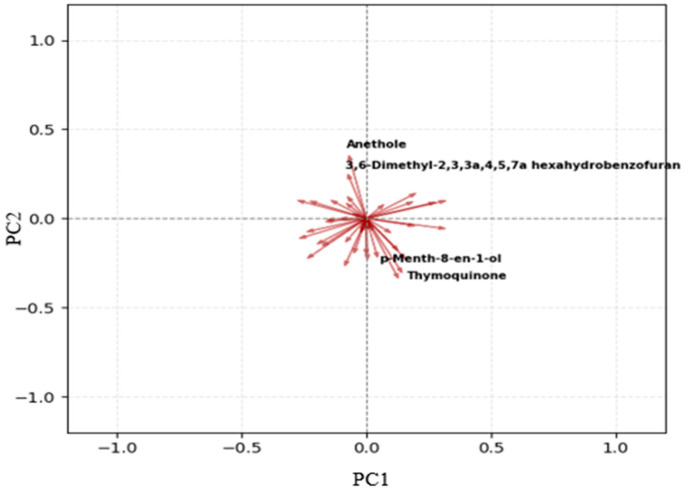
PCA loading plot showing the contributions of the 40 identified VOCs.

The PCA score plot presented in Figure 1 demonstrates a clear separation among the five honey samples originating from different botanical sources. The first principal component (PC1) explains 49.8% of the total variance, while the second principal component (PC2) accounts for 22.6%, together capturing the data variability. This differentiation is visually evident by the distinct clustering of the samples with minimal overlap, underscoring the robustness of PCA in distinguishing honey types based on their chemical or volatile profiles. Importantly, technical replicates from the same independent batch cluster tightly together (highlighted with connected markers), indicating low analytical variability and good method reproducibility under the automated HS-SPME-GC-MS conditions. The corresponding loading plot (Figure 2) reveals the volatile compounds responsible for this separation. Key chemotaxonomic markers exhibit prominent loadings: anethole shows a strong positive loading on PC1, aligning with the clustering of coriander and chehelgiah honeys; thymoquinone contributes significantly to the astragalus group positioning; p-menth-8-en-1-ol loads heavily on PC2, driving orange blossom distinction; and the bicyclic furan derivative (dill ester) influences rosemary separation.

Compounds exhibiting positive loadings on both PC1 and PC2 (upper-right quadrant of the loadings plot) are most influential in positioning the rosemary class, which occupies the positive–positive region in the score plot (Figure 1). These include dill ester (the primary rosemary marker with a strong positive contribution to PC1 and moderate positive to PC2), cedrol, 1-pentadecene, and 8-heptadecene. These volatiles, enriched in rosemary honey due to terpenoid and fatty acid pathways characteristic of Rosmarinus officinalis, collectively explain the distinct placement of rosemary samples in this quadrant.

Conversely, compounds with negative loadings on both PC1 and PC2 (lower-left quadrant) are key drivers of coriander separation, as this class clusters in the negative–negative region. Prominent examples include anethole (strong negative loading on PC1 and moderate negative on PC2, the signature phenylpropanoid of coriander), fenchone, (-)-carvone, and nonanoic acid. These compounds reflect the characteristic aromatic aldehyde, monoterpenoid ketone, and fatty acid profile of Coriandrum sativum nectar and are responsible for pulling coriander samples toward the negative–negative quadrant, underscoring their biochemical distinction from other classes.

These loadings confirm that PCA separation is biologically meaningful, driven by origin-specific VOCs identified in Table 1 and Table 2, and validate the robustness of HS-SPME-GC-MS volatile profiling for botanical authentication. The tight clustering of technical replicates from the same independent batch (highlighted with connected markers in Figure 1) further indicates low analytical variability and excellent method reproducibility under the automated extraction and analysis conditions.

Pourmoradian et al. detected honey adulteration with artificial sweeteners by distinguishing authentic honey using PCA [16]. Also, Akbari et al. (2020) determined the honey origin based on their floral components using PCA [17].

**Figure 3:**
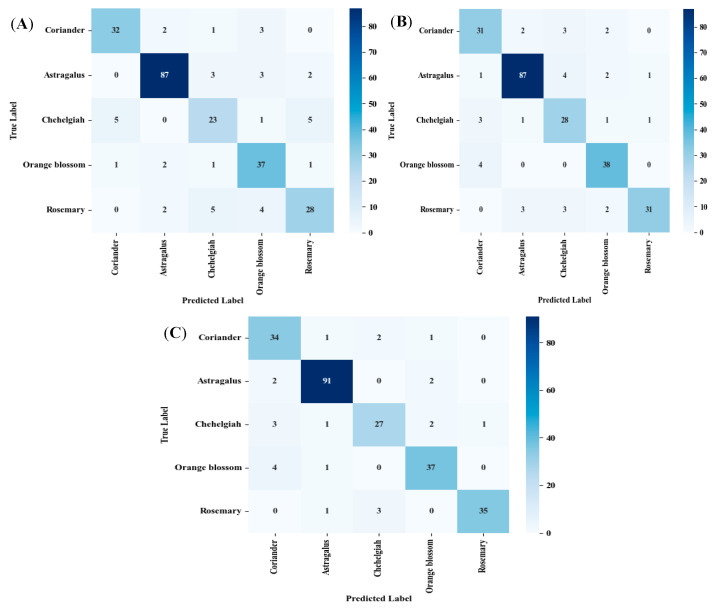
Confusion matrices: (**A**) RF, (**B**) XGBoost, and (**C**) NN.

The classification performance of the three supervised models is presented in Table 3. Overall accuracy ranged from 83.47% in RF to 90.32% in NN, with XGBoost achieving 86.69%, indicating robust discriminative capability across all models, particularly for a multiclass problem involving subtle physicochemical and sensory differences among honey types. Model performance metrics reported in Table 3 and confusion matrices (Figure 3A–C) were calculated exclusively on the independent test set (~six batches, 18 replicates), confirming generalization beyond the training data.

NN: As a powerful deep learning framework, an NN excels in automated feature extraction and classification of food and agricultural products based on geographical origin, cultivar, and quality attributes [18]. In this study, the NN outperformed the ensemble methods, attaining the highest overall accuracy (90.32%) and demonstrating superior balance across precision (0.79–0.97), recall (0.79–0.96), and F1-score (0.82–0.96). Notably, the NN achieved near-perfect precision (0.97) and F1-score (0.93) for rosemary honey, alongside high sensitivity (0.96) for astragalus, suggesting effective capture of class-specific patterns in spectral or compositional features. Specificity remained consistently high (>0.95) across all classes and models, reflecting strong negative class discrimination. Our result matched with Koraqi et al. (2025), who used an NN to discriminate the Kosovan honey botanical origin [19].

XGBoost: As another advanced ensemble technique, this approach constructs a robust predictive model by sequentially integrating multiple weak learners. It efficiently manages missing data and scales to large datasets through parallel computation [20]. Despite extensive adoption across fields including finance, healthcare, e-commerce, transportation, industry, and meteorology, its application in food origin traceability is still emerging [21]. The results in Table 3 show that XGBoost followed closely with 86.69% accuracy and exhibited excellent precision (≥0.94) for orange blossom, astragalus, and rosemary, indicating low false positive rates in these classes. Its recall and sensitivity were particularly strong for chehelgiah (0.82 and 0.83, respectively), outperforming RF in this challenging class. The model’s use of gradient boosting likely enhanced its ability to model complex, non-linear interactions among input features. In 2025, Simão et al. employed digital image processing integrated with machine learning algorithms to classify bee pollen samples according to species origin, attaining testing accuracies of up to 77% with XGBoost [22].

RF: Developed in the early 2000s, RF is a reliable and intuitive ensemble learning algorithm that aggregates outputs from numerous decision trees to mitigate overfitting and enhance predictive accuracy [23]. In the current study, RF, while achieving the lowest accuracy (83.47%), maintained balanced performance, with F1-scores ranging from 0.69 (chehelgiah) to 0.93 (astragalus). Its highest precision and sensitivity were observed for astragalus (0.94 and 0.91), consistent with the algorithm’s robustness to noisy or correlated features commonly encountered in food authentication datasets. Its lower performance for chehelgiah and rosemary suggests potential overlap in feature distributions, possibly due to biochemical similarity with co-occurring floral sources. In a recent study, Kantemiris et al. (2025) [24] demonstrated the efficacy of Laser-Induced Breakdown Spectroscopy (LIBS), coupled with machine learning algorithms, for the rapid and in situ authentication of honey’s botanical origin. By analyzing emission spectra and using RF, the approach successfully classified samples from eight distinct botanical sources [24].

Across all models, astragalus was the most accurately classified (F1-score ≥ 0.93), likely due to distinct pollen or phytochemical markers. In contrast, chehelgiah consistently showed the lowest metrics (F1-score: 0.69–0.82), indicating greater classification difficulty—possibly attributable to intraspecific variability or underrepresentation in the training data. High specificity across classes (>0.94) underscores the models’ reliability in ruling out incorrect botanical assignments, a critical requirement for honey authenticity verification.

Overall, the NN emerged as the most effective classifier for botanical honey discrimination, followed by XGBoost and RF. These results support the viability of machine learning, particularly deep learning architectures, for high-accuracy, multiclass food authenticity tasks when coupled with appropriate preprocessing and stratified sampling. Furthermore, the confusion matrices for all models, presented in Figure 3, provide a detailed visualization of classification performance across the five botanical honey classes. These matrices confirm the reliability of the reported accuracy, precision, recall, and F1-score values by illustrating the distribution of true positives, true negatives, and misclassifications. The predominance of diagonal elements—particularly prominent in the NN model—validates its superior discriminative ability, while minimal off-diagonal entries across all models affirm high specificity and low confusion between classes, reinforcing the robustness of the proposed machine learning framework for honey botanical origin authentication.

The comparative overview in Table 4 illustrates representative studies employing chromatographic techniques combined with chemometrics for honey botanical origin discrimination. These works highlight the effectiveness of volatile or metabolite fingerprinting, but most rely on traditional multivariate methods (e.g., PCA, OPLS-DA, LDA) or limited ML approaches. Our study builds upon this foundation by integrating HS-SPME-GC-MS volatile profiling with advanced supervised machine learning (RF, XGBoost, and NN), achieving higher multiclass accuracy (90.32% with NN) for five botanically diverse honeys and identifying key markers that align with or extend previous findings. This positions our workflow as a promising enhancement for rapid, reliable authentication in complex multiclass scenarios.

## 4. Discussion

The integration of HS-SPME with GC-MS in this study yields comprehensive VOC profiles that serve as discriminative fingerprints for honey botanical origins, effectively bridging analytical chemistry and advanced chemometrics. By extracting and quantifying 40 VOCs across 57 samples from coriander, orange blossom, astragalus, rosemary, and chehelgiah sources, we identified class-specific markers such as anethole (predominant in coriander and chehelgiah, contributing to anisic notes via phenylpropanoid pathways) and thymoquinone (hallmark of astragalus, linked to quinone biosynthesis and potential bioactivity). These markers not only validate floral fidelity but also highlight biochemical crosstalk between nectar sources and honey maturation, where enzymatic transformations (e.g., the oxidation of monoterpenes in rosemary leading to furan derivatives like dill ester) amplify origin-specific signatures. PCA visualization (PC1: 49.8%, PC2: 22.6%) underscores this chemical divergence, with tight clustering reflecting minimal intrasource variability and robust intersource separation, attributable to standardized extraction conditions (e.g., 60 °C agitation at 500 rpm) that minimize thermal artifacts and enhance reproducibility.

Supervised classification models further transform these fingerprints into actionable authenticity tools, with the NN achieving superior performance (90.32% accuracy) through its multilayer perceptron architecture, which excels in hierarchical feature abstraction from high-dimensional GC-MS data. This edge over XGBoost (86.69%) and RF (83.47%) arises from the NN’s ability to model non-linear interactions among correlated VOCs—such as co-occurring terpenoids in citrus-derived honeys—via backpropagation and ReLU activation, yielding elevated F1-scores (e.g., 0.96 for astragalus). In contrast, XGBoost’s gradient-boosting paradigm, with its tree-based regularization and handling of sparse features (e.g., absent compounds denoted by zeros), proves advantageous for noisy classes like chehelgiah (recall: 0.82), where multifloral overlaps introduce ambiguity. RF’s ensemble averaging mitigates variance but struggles with subtle gradients, as evidenced by lower sensitivity in rosemary (0.71), potentially due to unoptimized tree depth limiting resolution of biochemical similarities (e.g., shared phenolic precursors). A technical insight here is the value of incorporating SHAP (SHapley Additive exPlanations) values post-training to interpret model decisions: for instance, anethole and thymoquinone emerge as top contributors in NN predictions, offering explainable AI for regulatory audits and reducing black-box concerns in food forensics.

Benchmarking against contemporary works reveals our framework’s advancements in multiclass precision. Unlike Simão et al. (2025) [22], who attained ~77% accuracy in bee pollen speciation via XGBoost on image-derived features, our VOC-centric approach leverages molecular specificity to surpass 90% in a comparable five-class scenario, minimizing the environmental confounders inherent to visual data. Similarly, Kantemiris et al. (2025) [24] reported strong RF performance with LIBS spectra for eight honey origins, but our method’s lower instrumentation barrier—HS-SPME-GC-MS requires no laser ablation and supports portable variants—enhances deployability for field-based fraud detection. A noteworthy technical edge is the potential for transfer learning: pretraining NNs on larger public VOC datasets (e.g., from NIST libraries) could fine-tune for rare origins, boosting generalization amid global honey trade variability. Limitations include the absence of hyperparameter optimization (e.g., grid search for NN hidden layers or XGBoost learning rates), which might elevate accuracies further, and sample size constraints for underrepresented classes like rosemary, warranting ensemble strategies (e.g., stacking an NN with XGBoost) to mitigate bias.

This methodology not only addresses gaps in authenticating Iranian–Spanish honeys—where regional nectar diversity challenges traditional melissopalynology—but also paves the way for hybrid systems integrating GC-MS with emerging sensors (e.g., e-noses calibrated via our VOC markers) for rapid, cost-effective screening. By emphasizing volatile chemotaxonomy and interpretable ML, it fortifies supply chain integrity against sophisticated adulteration, fostering sustainable beekeeping and consumer trust in premium monofloral markets.

### Limitations

A key limitation of the present study is the relatively small number of independent batches per botanical class (three to four batches per origin, yielding 9–12 technical replicates after triplication), resulting in modest class numerosity for a five-class problem. This constraint is common in exploratory studies on monofloral honeys, where access to authenticated, batch-specific, pure samples from single floral sources is logistically and seasonally restricted, particularly for less commercially widespread origins such as chehelgiah and astragalus [12]. Although technical replicates were included to increase statistical power within each batch, the modest independent sample size per class increases the risk of model overfitting and limits the generalizability of the classification performance to broader populations. To mitigate these risks, we deliberately selected algorithms known for their robustness in small-sample, high-dimensional, and multiclass settings: RF and XGBoost benefit from ensemble mechanisms (bagging and regularized boosting, respectively) that inherently reduce variance and overfitting, while performing effectively with limited data and handling potential class imbalance through inherent mechanisms or stratified sampling [30]. The NN (MLPClassifier with a single hidden layer of 100 neurons) was included as a proof-of-concept comparison to evaluate deep learning potential, but we intentionally avoided extensive hyperparameter tuning or architectural complexity to prevent over-optimism and reduce the risk of overfitting on small data. All models were trained with stratified 70/30 train–test splitting at the independent batch level to avoid data leakage and ensure fair evaluation. Despite these safeguards, the reported accuracies (83.47–90.32%) should be interpreted cautiously as indicative of method feasibility in an exploratory context rather than definitive performance on large-scale datasets. Future validation on larger, more diverse cohorts will be essential to confirm robustness and generalizability.

## 5. Conclusions

As a preliminary and exploratory study with limited independent batches per botanical class (three to four per origin), this work demonstrates the feasibility of HS-SPME-GC-MS volatile profiling combined with advanced machine learning (RF, XGBoost, and NN) for multiclass botanical authentication of honeys. The achieved classification accuracies (83.47–90.32%) are encouraging in this constrained setting, but the modest sample size and class numerosity necessitate cautious interpretation and further validation on larger, more diverse cohorts. Future studies should expand sample diversity, optimize model hyperparameters, and explore multi-modal data integration to enhance robustness and support broader regulatory and industrial applications.

## Figures and Tables

**Table 1:** Profile of honey volatile compounds identified using GC-MS analysis.

RT ^1^	Volatile Compounds	Chemical Class	Kovat Index (KI) ^2^	
			Exp.	Lit.
6.844	Nonane	Alkane	900	900
8.929	Benzaldehyde	Aromatic aldehyde	964	961
10.554	Octanal	Aldehyde	1008	1000
11.682	D-Limonene	Monoterpene	1028	1030
12.211	Benzeneacetaldehyde	Aromatic aldehyde	1047	1045
12.888	2-Octenal, (E)-	Unsaturated aldehyde	1065	1062
13.481	1-Octanol	Alcohol	1082	1076
14.245	Fenchone	Monoterpenoid ketone	1086	1088
14.777	Linalool	Monoterpenoid alcohol	1098	1098
14.976	Nonanal	Aldehyde	1103	1102
15.246	Phenylethyl alcohol	Aromatic alcohol	1109	1110
16.627	p-Menth-8-en-1-ol, stereoisomer	Monoterpenoid alcohol	1141	1146
16.991	Bicyclo [3.1.0] hexan-3-ol, 4-methyl-1-(1methylethyl)-	Monoterpenoid alcohol	1149	1155
18.068	1-Nonanol	Alcohol	1173	1172
18.714	dill ester	Furan derivative (sesquiterpenoid-like)	1187	1192
19.238	Estragole	Phenylpropanoid	1200	1196
19.650	Decanal	Aldehyde	1203	1203
20.125	3-Cyclohexene-1-acetaldehyde,.alpha.,4-dimethyl-	Monoterpenoid aldehyde	1214	1217
21.308	(-)-Carvone	Monoterpenoid ketone	1239	1240
22.196	2-Decenal, (E)-	Unsaturated aldehyde	1258	1264
22.524	Nonanoic acid	Fatty acid	1267	1272
22.698	1-Decanol	Alcohol	1269	1272
23.341	Anethole	Phenylpropanoid	1285	1284
23.459	Thymol	Monoterpenoid phenol	1287	1287
23.460	p-Mentha-1,8-dien-7-ol	Monoterpenoid alcohol	1287	1295
25.568	Methyl anthranilate	Aromatic ester	1334	1341
26.223	Naphthalene, 1,2-dihydro-1,1,6-trimethyl-	Sesquiterpene derivative	1348	1355
28.436	Tetradecane	Alkane	1398	1400
28.731	Tetradecanal	Aldehyde	1405	1409
30.311	5,9-Undecadien-2-one, 6,10-dimethyl-	Sesquiterpenoid ketone	1443	1435
32.296	1-Pentadecene	Alkene	1498	1492
34.118	.alpha.-Calacorene	Sesquiterpene	1535	1542
36.625	Hexadecane	Alkane	1598	1600
36.769	Cedrol	Sesquiterpenoid alcohol	1603	1596
39.522	8-Heptadecene	Alkene	1676	1680
42.483	Octadecanoic acid	Fatty acid	1757	1761

^1^ Retention Time (min). ^2^ KI: (Exp.) = experimental Kovats index; (Lit.) = literature Kovats index (using NIST libraries).

**Table 2:** Main volatile components of different botanical honey samples identified by GC-MS (%).

Volatile Compounds	RT (min)	Coriander	Astragalus	Chehelgiah	Orange blossom	Rosemary
Nonane	6.844	3.38 (3.38 ± 0.12)	–	2.20 (2.20 ± 0.09)	2.24 (2.24 ± 0.11)	2.80 (2.80 ± 0.15)
Benzaldehyde	8.929	–	–	–	1.79 (1.79 ± 0.08)	–
Octanal	10.554	1.71 (1.71 ± 0.10)	9.42 (9.42 ± 0.25)	–	–	–
Benzeneacetaldehyde (phenylacetaldehyde)	12.211	–	–	0.33 (0.33 ± 0.04)	1.19 (1.19 ± 0.07)	–
1-Octanol	13.481	11.99 (11.99 ± 0.45)	–	–	–	–
Fenchone	14.245	2.31 (2.31 ± 0.14)	–	–	–	–
Linalool	14.777	1.93 (1.93 ± 0.09)	6.08 (6.08 ± 0.22)	–	1.27 (1.27 ± 0.06)	–
Nonanal	14.976	17.54 (17.54 ± 0.68)	9.86 (9.86 ± 0.41)	2.42 (2.42 ± 0.13)	4.86 (4.86 ± 0.19)	4.00 (4.00 ± 0.17)
p-Menth-8-en-1-ol	16.627	–	–	–	3.64 (3.64 ± 0.16)	–
Sabinene hydrate	16.991	–	2.40 (2.40 ± 0.12)	–	6.13 (6.13 ± 0.28)	–
1-Nonanol	18.068	1.02 (1.02 ± 0.05)	–	–	1.73 (1.73 ± 0.08)	1.03 (1.03 ± 0.06)
Dill ester	18.714	2.10 (2.10 ± 0.11)	1.78 (1.78 ± 0.09)	–	–	3.32 (3.32 ± 0.18)
Decanal	19.65	8.24 (8.24 ± 0.32)	2.35 (2.35 ± 0.12)	2.66 (2.66 ± 0.14)	1.14 (1.14 ± 0.07)	–
Perillaldehyde	20.125	–	2.09 (2.09 ± 0.10)	–	3.75 (3.75 ± 0.17)	–
(-)-Carvone	21.308	4.77 (4.77 ± 0.21)	–	–	–	–
Thymoquinone	21.56	–	8.17 (8.17 ± 0.38)	–	–	–
Nonanoic acid	22.524	–	–	1.36 (1.36 ± 0.07)	–	–
Anethole	23.341	3.84 (3.84 ± 0.18)	–	2.11 (2.11 ± 0.10)	–	–
Naphthalene, 1,2-dihydro-1,1,6-trimethyl-	26.223	–	–	2.45 (2.45 ± 0.13)	1.20 (1.20 ± 0.06)	–
Tetradecane	28.436	–	–	12.94 (12.94 ± 0.52)	–	–
1-Pentadecene	32.296	–	–	–	1.36 (1.36 ± 0.08)	1.90 (1.90 ± 0.11)
Hexadecane	36.625	3.67 (3.67 ± 0.16)	–	2.56 (2.56 ± 0.12)	–	–
Cedrol	36.769	–	–	–	–	1.65 (1.65 ± 0.09)
8-Heptadecene	39.522	1.04 (1.04 ± 0.05)	–	–	2.11 (2.11 ± 0.10)	2.62 (2.62 ± 0.14)
Octadecanoic acid (stearic acid)	42.483	–	–	–	1.44 (1.44 ± 0.07)	2.26 (2.26 ± 0.12)

Chemical components present at concentrations below one percent are not listed in the Table.

**Table 3:** Comparative performance of classification models.

Model	Evaluation Metric	Samples				
		Coriander	Orange Blossom	Astragalus	Rosemary	Chehelgiah
RF	Precision	0.84	0.77	0.94	0.78	0.70
	Recall	0.84	0.88	0.92	0.72	0.68
	F1-score	0.84	0.82	0.93	0.75	0.69
	Sensitivity	0.84	0.88	0.91	0.71	0.67
	Specificity	0.97	0.94	0.96	0.96	0.95
	Accuracy = 83.47%					
XGBoost	Precision	0.79	0.94	0.94	0.94	0.74
	Recall	0.82	0.90	0.92	0.79	0.82
	F1-score	0.81	0.87	0.93	0.86	0.78
	Sensitivity	0.81	0.90	0.91	0.79	0.83
	Specificity	0.96	0.96	0.96	0.99	0.95
	Accuracy = 86.69%					
NN	Precision	0.79	0.88	0.96	0.97	0.84
	Recall	0,89	0.88	0.96	0.90	0.79
	F1-score	0.84	0.88	0.96	0.93	0.82
	Sensitivity	0.89	0.88	0.95	0.89	0.79
	Specificity	0.95	0.97	0.97	0.99	0.97
	Accuracy = 90.32%					

**Table 4:** Several studies on honey botanical discriminations.

Technique/Methods	Chemometrics Model	Key Findings	Reference
Physicochemical parameters	LDA, ANN, PCA	High-accuracy classification of honeys according to botanical origin	[25]
HS-GC-IMS	OPLS-DA	Identification of volatile compounds for botanical characterization	[26]
LC-QTOF-MS	RF, PLS-DA	Rapid and reliable classification using metabolomic fingerprinting	[5]
NIR	PCA	Identification of botanical markers enabling varietal honey discrimination	[27]
Electronic Nose and SPME-GC-MS	PCA	Successful volatile profile discrimination of botanical origin	[28]
HS-GC-IMS	PCA	Effective detection of carbohydrate markers to detect adulteration	[29]
HS-SPME-GC-MS	PCA-RF-XGBoost-NN	Rapid and reliable classification of five different botanical honey samples	Our study

## Data Availability

The original contributions presented in the study are included in the article/Supplementary Materials. Further inquiries can be directed to the corresponding author.

## Supplementary Materials

The following supporting information can be downloaded at: https://www.mdpi.com/article/10.3390/foods15020389/s1, Table S1: Detailed information on honey samples.

## Abbreviations

GC–MS	Gas Chromatography–Mass Spectrometry
HS-SPME	Headspace Solid-Phase Microextraction
VOC	Volatile Organic Compound
PCA	Principal Component Analysis
RF	Random Forest
XGBoost	eXtreme Gradient Boosting
NN	Neural Network
PC1	Principal Component 1
PC2	Principal Component 2
