# Supplementary material for: Honey Botanical Origin Authentication Using HS-SPME-GC-MS Volatile Profiling and Advanced Machine Learning Models (Random Forest, XGBoost, and Neural Network)
Source: Foods. 2026 Jan 21;15(2):389. doi: 10.3390/foods15020389 (PMC12841070; doi:10.3390/foods15020389)
Supplement: Supplementary file 1 [file foods-15-00389-s001.zip › foods-4100293-supplementary.pdf]

Table S1: Detailed information on honey samples

Batch ID	Botanical Variety	Country	Region/Province	Latitude (°N)	Longitude (°E)	Harvest Season
C1–C4	Coriander (Coriandrum sativum)	Iran	Tehran/Esfahan	35.7	51.4	Summer 2025
O1–O4	Orange blossom (Citrus sinensis/aurantium)	Spain	Valencia/Alicante	38.3–38.5	-0.5–0.8	Spring 2025
A1–A4	Astragalus (Astragalus spp.)	Iran	Lorestan/Kermanshah	33.5–34.5	46.0–48.0	Summer 2025
R1–R3	Rosemary (Rosmarinus officinalis)	Spain	Alicante/Murcia	38.0–38.2	-1.0–0.5	Spring 2025
Ch1–Ch4	Chehelgiah (local Iranian wildflower, predominantly from Thymus spp. and other Lamiaceae)	Iran	Kurdistan	35.0–36.0	46.5–47.5	Summer 2025
